# Modeling of the potential geographical distribution of naked oat under climate change

**DOI:** 10.3389/fpls.2022.1009577

**Published:** 2023-01-12

**Authors:** Mingxing Qin, Xinyue Gao, Meichen Feng, Ning Jin, Chao Wang, Wenjuan Cheng

**Affiliations:** ^1^ College of Resources and Environment, Shanxi Agricultural University, Taigu, Shanxi, China; ^2^ College of Plant Protection, Shanxi Agricultural University, Taigu, Shanxi, China; ^3^ State Key Laboratory of Sustainable Dryland Agriculture (in preparation), Shanxi Agricultural University, Taiyuan, Shanxi, China; ^4^ College of Agronomy, Shanxi Agricultural University, Taigu, Shanxi, China; ^5^ Department of Resources and Environmental Engineering, Shanxi Institute of Energy, Jinzhong, Shanxi, China; ^6^ Tianjin Academy of Agricultural Sciences, Tianjin, China

**Keywords:** climate change, distribution, naked oat, North China, Maxent

## Abstract

**Introduction:**

Naked oat (*Avena sativa L.)*, is an important miscellaneous grain crop in China, which is rich in protein, amino acids, fat and soluble dietary fiber. The demand for functional foods is gradually increasing as living standards rise, and the output of minor cereals in China is increasing annually. The planting layout of naked oat is scattered and lacks planning, which seriously restricts the development of the naked oat industry. The increase in miscellaneous grain production will not only be impacted by cultivation methods and management techniques, but the potential impact of global climate change needs to be considered. North China is the main area for naked oat production, worldwide.

**Methods:**

In this study, the potential distribution range of naked oat in North China was forecast based on historical distribution data and the Maxent model under climate change conditions. The performance of the model was relatively high.

**Results:**

The results indicated that the most suitable area for the potential geographic distribution of naked oat in North China was 27.89×104 km^2^, including central and northeastern Shanxi, and northeastern and western Hebei and Beijing, gradually moving northward. The core suitable area increased, and the distribution of naked oat had an obvious regional response to climate warming; the main environmental factors affecting the potential geographic distribution were precipitation factor variables (precipitation seasonality (variation coefficient)), terrain factor variables (elevation) and temperature factor variables (temperature seasonality (Standard Deviation*100)).

**Discussion:**

In this study, the Maxent model was used to analyze and predict suitable areas for naked oat in North China, and the distribution of suitable areas was accurately divided, and the main climatic factors affecting the distribution of naked oat were identified. This research provides data support and theoretical support for the optimal planting zone of naked oat in North China.

## Introduction

The relationship between the dynamic changes in vegetation and climate has been a hot issue in global change and biogeography research (Kaufmann et al., 2003; Bellard et al., 2012; Bonan and Doney, 2018). Climate is the most important environmental factor affecting the distribution of species and vegetation at the regional and global scale, and climate change has a huge impact on biodiversity and species distribution areas (Jiang, 2013; Giorgi, 2019). The Intergovernmental Panel on Climate Change (IPCC) has reported for nearly 130 years that the global average surface temperature has risen by 0.85°C and the average surface temperature is likely to rise continuously in the future (Stocker, 2013). Also, the changes in global climate will alter precipitation patterns and affect the distribution patterns of species (Williams et al., 2007; Huang et al., 2013; Cheng et al., 2016). Therefore, it is an important guide for the formulation of biodiversity conservation strategies to study the responses of the potential geographical distribution of species to the future climate change and predict the changes of the potential geographical distribution of species in the context of future climate change.

Naked oat (Avena sativa L.) is widely recognized as one of the highest nutritional cereal crops in the world. China is one of the birthplaces of naked oats in the world. And the yield and nutritional quality of naked oats in North China are significantly higher than those in other parts of China (Zhou, 2014). It has the advantages of cold tolerance, drought resistance, tolerance to infertile land, moderate salinity, and low agricultural risk factors, and the 35–60°N geographical zone is a suitable planting area for naked oat (Su et al., 2007; Zhao et al., 2007). Northwestern China, along the Great Wall, is suitable for planting naked oat. The planting area accounted for 16% of the grain sown area, and the production accounted for 9% of the total output. The planting area is predominantly in the central and western area of China, mainly in Shanxi, Hebei, Inner Mongolia and other provinces (Hu, 2007; Bo et al., 2021). With the improvement in social and economic development and people’s living standards, the planting areas of naked oat have expanded from the traditional ecological suitable areas to non-traditional production areas, and the planting areas continue to increase. At present, there are few studies on the potential geographical distribution areas of naked oat. In addition, studies on the spatial distribution of native populations of naked oat are limited to establishing sample plots and analyzing the spatial pattern of individuals in a certain area. There are few studies to predict the spatial distribution patterns of naked oat under future climatic conditions (Nian et al., 2020). The planting layout of naked oat is scattered and lack of planning, which seriously restricts the development of naked oat industry. The miscellaneous grain production will be affected not only by farming methods and management techniques, but also by global climate change. Therefore, it is necessary to predict the potential distribution of naked oats and form scientific regional planning, which helps for the formulation of management strategies and policy support for farmers to plant naked oats.

Species distribution models have been widely used to study the potential geographic distribution of species under climate conditions. The models use temperature, relative humidity, precipitation, elevation and other environmental factors to predict the potential geographic distribution of species (Phillips and Miroslav, 2008; Wiens et al., 2009; Li et al., 2013; Li et al., 2015; Zhang et al., 2020; Zurell et al., 2020). The Maximum Entropy Model (Maxent) is a machine learning model that estimates the probable distribution of targets by calculating the probable distribution of maximum entropy (Zhang, 2015). Compared with other models, the Maxent model can still obtain high prediction accuracy in situations where only species distribution data are required (Kumar, 2009). With the in-depth development of the intersection of conservation biology, ecology and biogeography, the Maxent has been frequently applied to different species, different periods and different situations with different needs (Zhou et al., 2016; Ma and Sun, 2018; Perkins-Taylor and Frey, 2020). Elith et al. (2006) used 16 niche models to predict the potential geographical distribution of 226 species, and the results showed that the prediction result of the Maxent model was more accurate than that of other niche models. Chen et al. (2019) studied the habitat suitability of *Leymus chinensis* based on the Maxent model, analyzed the suitable climatic characteristics of *Leymus chinensis* using bioclimatic data, and obtained the geographical distribution range of this species and the bioclimatic factors that dominated its distribution. Wu et al. (2018) used the Maxent to predict the suitable habitat distribution area and the dominant climatic factors affecting the distribution of *Medicago ruthenica* under the background of climate change.

During the development of grain production in China, in order to pursue high grain yield and solve the problem of grain security, the main focus is on the production of saple grains, while the role of small miscellaneous grains in agricultural production is ignored. Planting naked oat can not only provide nutritious and healthy food resources, but also help to achieve reasonable crop rotation, land use and soil fertilization, and it has a good application in pest control and reduction of chemical fertilizer and pesticide application, and is conducive to ensuring food safety (Bo et al., 2021). This can protect groundwater resources and contribute to the long-term stability of food or agricultural production (Yang, et al., 2022).

In this study, we predicted the potential geographic distribution changes of naked oat under different climatic conditions based on the Maxent model and historical distribution sites. The objectives of the current study were as follows: 1) simulate the potential geographic distribution range of naked oat in North China under climate change conditions; 2) investigate the major climatic variables that constrain the potential distribution of naked oat; 3) provide a theoretical reference framework for farmers and policy-making for future planting of naked oat.

## Materials and methods

### Data sources and processing

#### Data on the distribution of naked oat

The distribution data on naked oat used in this study were obtained from the following sources: China Digital Herbarium (CVH).^1^ China National Herbarium Resource Sharing Platform,^2^ and Plant Photo Bank of China (PPBC).^3^ A total of 930 records of naked oat were collected. Duplicate occurrences and potential errors in distribution data were carefully checked and excluded. The record distribution points of naked oats in different places are greatly duplicated, because naked oats are planted in pieces, the planting sites are relatively dense, and the distance between the distribution points is relatively close.

**Figure 1:**
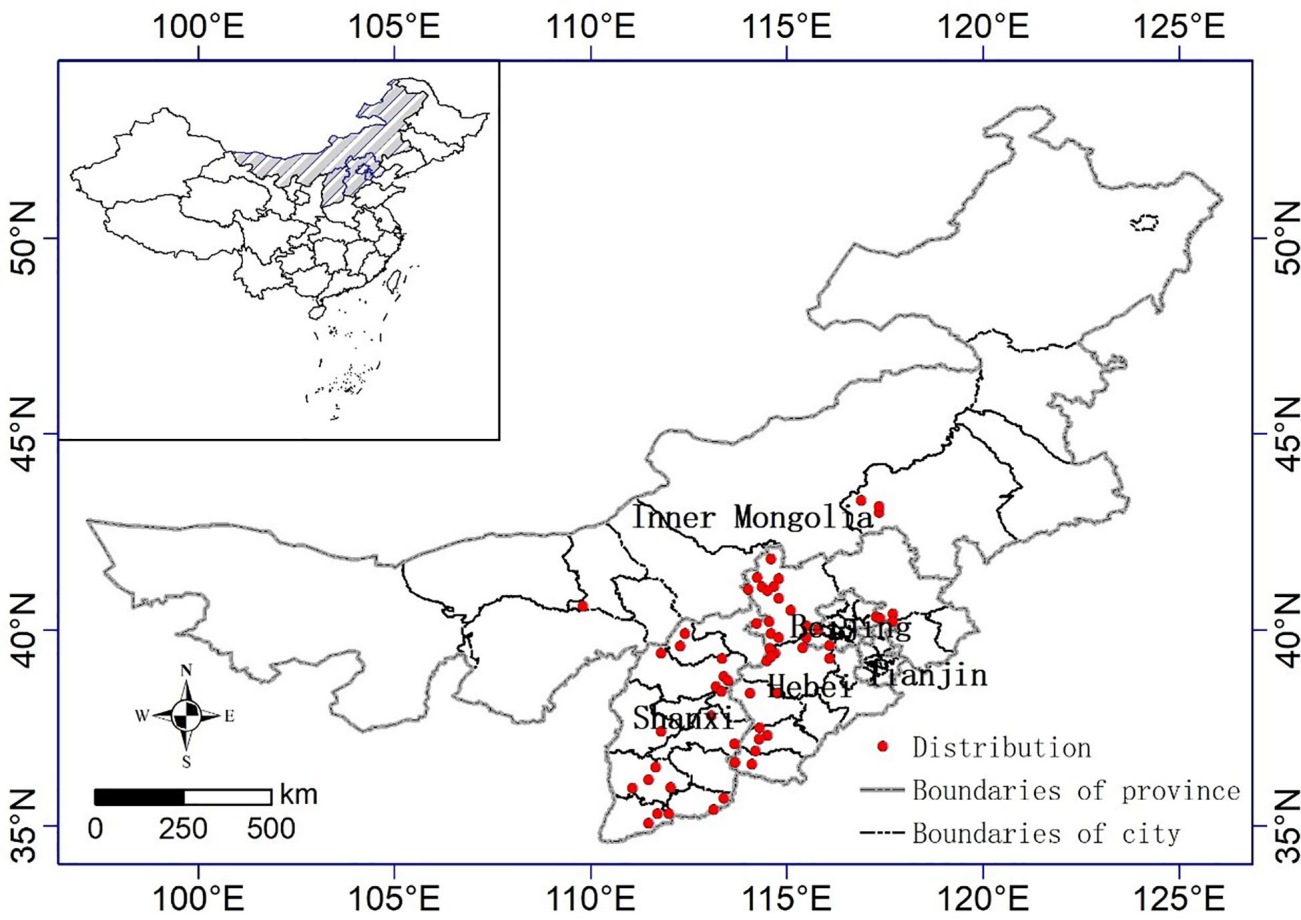
Distribution of naked oat in North China.

Thus, distribution site data can significantly influence the outcome of a model due to spatial autocorrelation. In order to reduce spatial autocorrelation and sample bias among occurrence data, A resolution (a grid cell size of 1 × 1 km^2^ ) was generated and a single point was randomly selected from each cell that included one or more sampling points. After removing duplicate and unclear geographical locations, 59 distribution records were retained. The maps of the locations for naked oats used in this study are presented in **Figure 1**.

#### Data on environmental variables

The current climate data (1990~2000) and future climate data (2021~2100) used in this study were derived from the World Climate Data website^4^ with a spatial resolution of 2.5 arc min. According to WorldClim, the spatial resolutions of the environmental variable layers are divided into 10 arc min, 5 arc min, 2.5 arc min, and 30 arc sec. This study was developed based on the 2.5 arc min spatial resolution layers. The results based on the analysis of the resolution of 30 arc sec and 2.5 arc min did not show observable differences, which was the same as the results of a previous study (Guisan et al., 2007). Compared to the data of 30 arc sec data, the processing is also much more rapid while employing 2.5 arc min data. The data of the resolution of 2.5 arc min were used for the previous studies to estimate the potential distribution of species (Jiang et al., 2022). We randomly selected three GCM (General Circulation Model) in the sixth phase of the International coupled Model comparison Program (CMIP6): the medium Resolution Climate system Model (BCC-CSM2-MR) released by the second Generation National (Beijing) Climate Center. The model includes four shared socio-economic pathways (SSPs) from the IPCC 6th Emissions Report (Zhang et al., 2020), SSP5-8.5: high forcing scenario with radiative forcing stabilized at 8.5 W/m^2^ in 2100; SSP3–7.0: medium to high forcing scenario with radiative forcing stabilized at 7.0 W/m^2^ in 2100; SSP2–4.5: medium forcing scenario with radiative forcing stabilized at 4.5 W/m^2^ in 2100; SSP1–2.6: low forcing scenario with radiative forcing stabilized at 2.6 W/m^2^ in 2100. In this study, 19 of these bioclimatic variables were selected based on a resolution of 2.5. The variables included annual mean temperature, minimum temperature of the coldest month, temperature seasonality, annual precipitation, precipitation in the driest period, and another 19 climate factors (**Table 1**). In addition, the altitude factor was also downloaded from the World Climate Data website, and two terrain factors, slope and aspect, were extracted from altitude using QGIS3.12.2. The soil data were derived from the HWSD.^5^ According to the relevant literature, the organic carbon content (t_oc) of the upper soil attribute in the soil variable and the organic carbon content (s_oc) and exchangeable sodium salt (s_esp) of the lower soil attribute were selected (Huang et al., 2022; Shao et al., 2022). In order to avoid overfitting of the model, principal component analysis was used to screen the environmental variables with low correlation but high significance (Fan et al., 2020). In this study, SPSS was used for Pearson correlation analysis of environmental factors, from which 9 environmental variables: bio2,bio4, bio13, bio14, bio15,Altitude, Slope, Subsoil Organic Carbon(s_oc), Subsoil Sand Fraction (s_esp) were selected.

**Table 1:** Environmental factors.

Indicator item	Description	Unit
Bio-1	Annual mean temperature	°C
Bio-2	Mean diurnal range	°C
Bio-3	Isothermality(Bio2/Bio7)(*100)	×100
Bio-4	Temperature seasonality (*100)	×100
Bio-5	Max temperature for warmest month	°C
Bio-6	Min temperature for coldest month	°C
Bio-7	Temperature annual range(Bio5-Bio6)	°C
Bio-8	Mean temperature of wettest quarter	°C
Bio-9	Mean temperature of driest quarter	°C
Bio-10	Mean temperature of warmest quarter	°C
Bio-11	Mean temperature of coldest quarter	°C
Bio-12	Annual precipitation	mm
Bio-13	Precipitation of wettest month	mm
Bio-14	Precipitation of driest month	mm
Bio-15	Precipitation seasonality	
Bio-16	Precipitation of wettest quarter	mm
Bio-17	Precipitation of driest quarter	mm
Bio-18	Precipitation of warmest quarter	mm
Bio-19	Precipitation of coldest quarter	mm
Altitude		m
Slope		°
S_esp	Subsoil Sand Fraction	% wt
T_OC	Topsoil Organic Carbon	% weight
S_OC	Subsoil Organic Carbon	% weight

#### Vector data

The geographic data were downloaded from the National Basic Geographic Information System^6^ as a 1:4 million vector map of China’s administrative divisions as the base map for the analysis. ArcGIS software version 10.5 and the Maxent V3.4.1 model^7^ were used in this study.

#### Model specification

**Figure 2:**
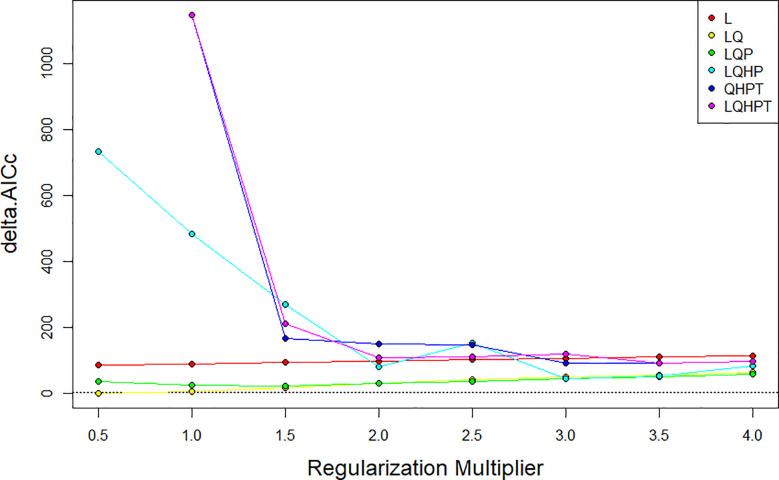
AICc value of parameter combinations based on the ENMeval calculation.

The Maxent model is a mathematical calculation method. This model can infer the unknown probable distribution of a species depending on its realistic distribution information and environmental variables. We can then obtain the potential distribution area of the target species by counting the distribution points of the study species based on the maximum entropy principle (Parmesan, 2006; Zhou et al., 2016). The geographic distribution data and environmental variable data of naked oat were imported into the Maxent software. In general, the default settings of the Maxent software produce an overfitted model. Therefore, we used feature type FC and regulated frequency doubling RM to optimize the model. The feature type represents different transformations (Lu et al., 2020) of the covariable, including linear (L), product (P), hinge (H), threshold (T) and quadratic feature (Q) (Elith et al., 2011). Adjusting the frequency doubling RM can reduce overfitting of the model and make the model smoother. We used the R package “ENMeval” to test whether the parameters were overfitted, and chose the combination of multipliers and feature classes based on these results (Qin et al., 2015). RM values ranged from 0.5 to 4.0 (increments of 0.5), and FC had 6 different combinations (L, LQ, LQP, LQHP, QHPT, LQHPT). We used the “Checkerboard2” method to calculate the Akaike information standard factor (AICc) and selected the lowest incremental AICc to run the final Maxent model (Wei et al., 2020). In this study, the best parameter setting for FC was 0.5 for LQ and RM (**Figure 2**) (Fan et al., 2020). The other parameters were set as follows: 25% of the distribution points were selected as the test set, 75% of the distribution points were used as the training set (Constandinou et al., 2018; Wang et al., 2021), thhe cross-validation method was used, the default setting of the maximum number of iterations was 500, the maximum number of background points was 10,000, and the rest of the default settings were selected, and the final output ASCII result file was the average of 10 iterations.

### Classification of naked oat habitat suitability classes

The result of the Maxent estimation was imported into ArcGIS10.5 software. Moreover, this study used the reclassify in spatial analysis tools, and classified the habitat suitability into four classes based on the natural breaks grading method (Jenks’ natural breaks). The default classification result of ArcGIS10.5 software was used to finally divide it into the unsuitability zone (0~0.08), low suitability zone (0.08~0.29), high suitability zone (0.29~0.52) and optimum suitability zone (0.52~0.96) (Ning et al., 2018). The potential geographical distribution area of naked oat in North China was established according to the suitability index. The same ranking was used to classify the suitability index of potential distribution areas of naked oat in the future.

### Evaluation of model accuracy

At present, many indicators are used to evaluate the Maxent model, including the area under the receiver operating characteristic curve (AUC), Kappa statistics (kappa), real skill statistics (TSS) and some AUC (pAUC) (Liu, 2014; Shabani et al., 2018). Among them, AUC is the most widely utilized, but it has many shortcomings. For example, it can neither provide information regarding the spatial distribution of model errors, nor measure omissions and delegation errors equally. In order to solve these problems, this study used pAUC to evaluate the performance of the model. This metric gives priority to missed errors over delegated errors, and can take into account the number of known or estimated errors in the event. In this study, pAUC was calculated using the Niche Toolbox^8^, the number of iterations was 1000, and the error (E) was 0.05.

## Results

### Model evaluation

**Figure 3:**
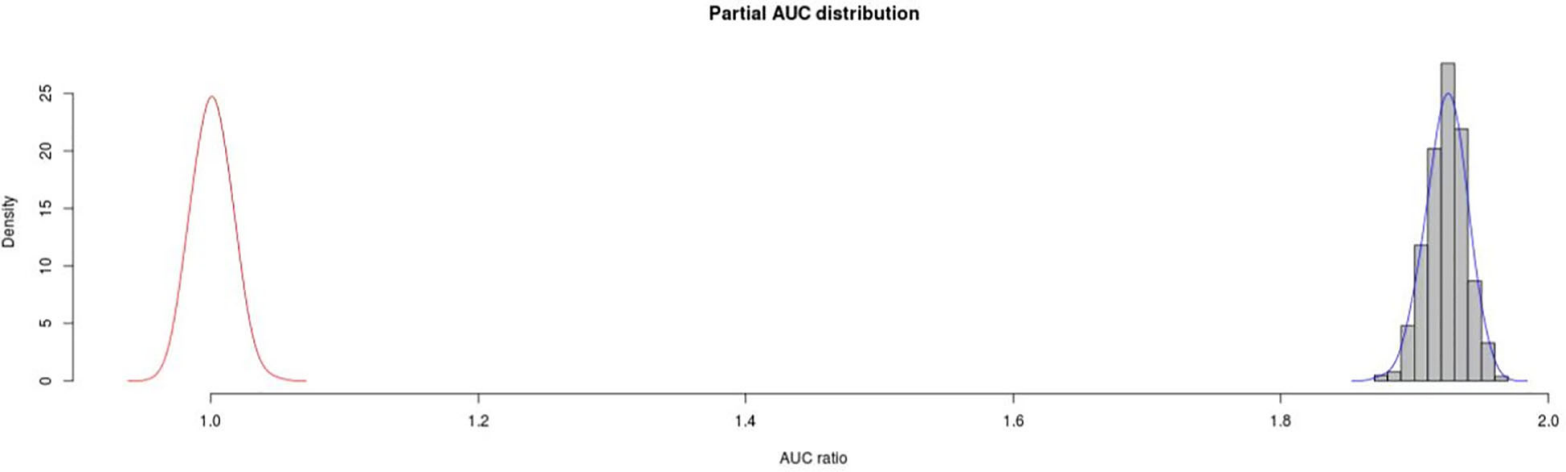
The results of pAUC.

The results showed that the pAUC value (in Epist 0.05) was 0.9618784, which indicated the good credibility of the model. In addition, the distribution of the AUC ratio calculated by AUC partial/AUC random was significantly larger than that of the random AUC ratio (P < 0.001), which indicated that the model had high performance (**Figure 3**).

### Analysis of factors affecting potential geographical distribution of naked oat

Among the environmental factor variables used for the Maxent model prediction (**Table 2**), the environmental factor variables with a higher contribution rate were: precipitation seasonality (coefficient of variation) (Bio15, 29.52%), altitude (25.76%), and temperature seasonality (standard deviation *100) (Bio4, 11.95%), with a cumulative contribution of 67.23%. Displacement importance values (displacement importance values are values that randomly displace each environmental factor variable on the training and background data). Larger values indicate a stronger dependence of the model on that variable The top three environmental factor variables were altitude (39.58%), temperature seasonality (standard deviation*100) (Bio4, 21.40%), and precipitation seasonality (coefficient of variation) (Bio15, 16.09%), with a cumulative value of 77.07%.

**Table 2:** Contribution rate of environmental variables and importance of replacement.

Code	Factor	Contribution/%	Permutation Importance/%
Bio15	Precipition Sensonality (Coefficient of Varitation)	29.52	16.09
Altitude	Altitude	25.76	39.58
Bio4	Temperature Seasonality (standard deviation*100)	11.95	21.4
Slope	Slope	7.72	0.13
Bio13	Precipition of Wettest Month	7.62	6.48
S_oc	Subsoil Organic Carbon	7.22	5.61
Bio14	Precipition of Driest Month	5.88	9.78
S_esp	Subsoil Sand Fraction	3.57	0.81
Bio2	Mean Diumal Range (Mean of Monthly(max temp-min temp))	0.76	0.13

**Figure 4:**
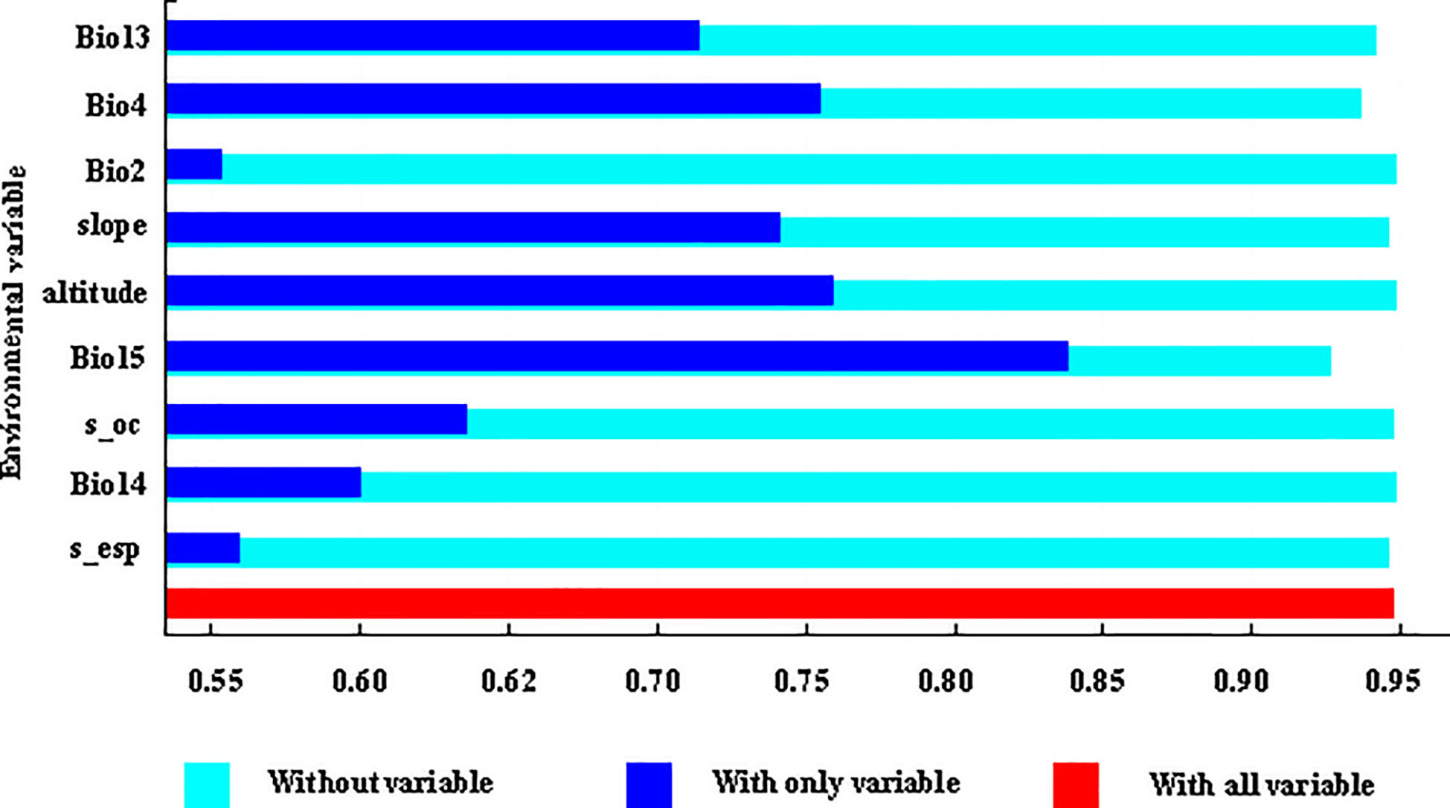
Verification results of the naked oat environmental factor knife cutting method.

From the results of the Jackknife test (**Figure 4**), it can be seen that when only a single environmental factor variable was used, the environmental factor variables that had the greatest impact on the regularized training gain were the seasonal variation coefficient of precipitation (Bio15), altitude, and temperature seasonality (Bio4), indicating that these environmental factor variables contain information that other environmental factor variables do not. On the whole, the main factors affecting the potential geographic distribution of naked oat under the current climate conditions were: precipitation factor variables, terrain factors and temperature factor variables. The relationship between the existence probability of naked oat and environmental factors can be judged according to the response curve of environmental factors. When the existence probability of naked oat was greater than 0.5, the corresponding environmental factor value was favorable for the growth of naked oat.

**Figure 5:**
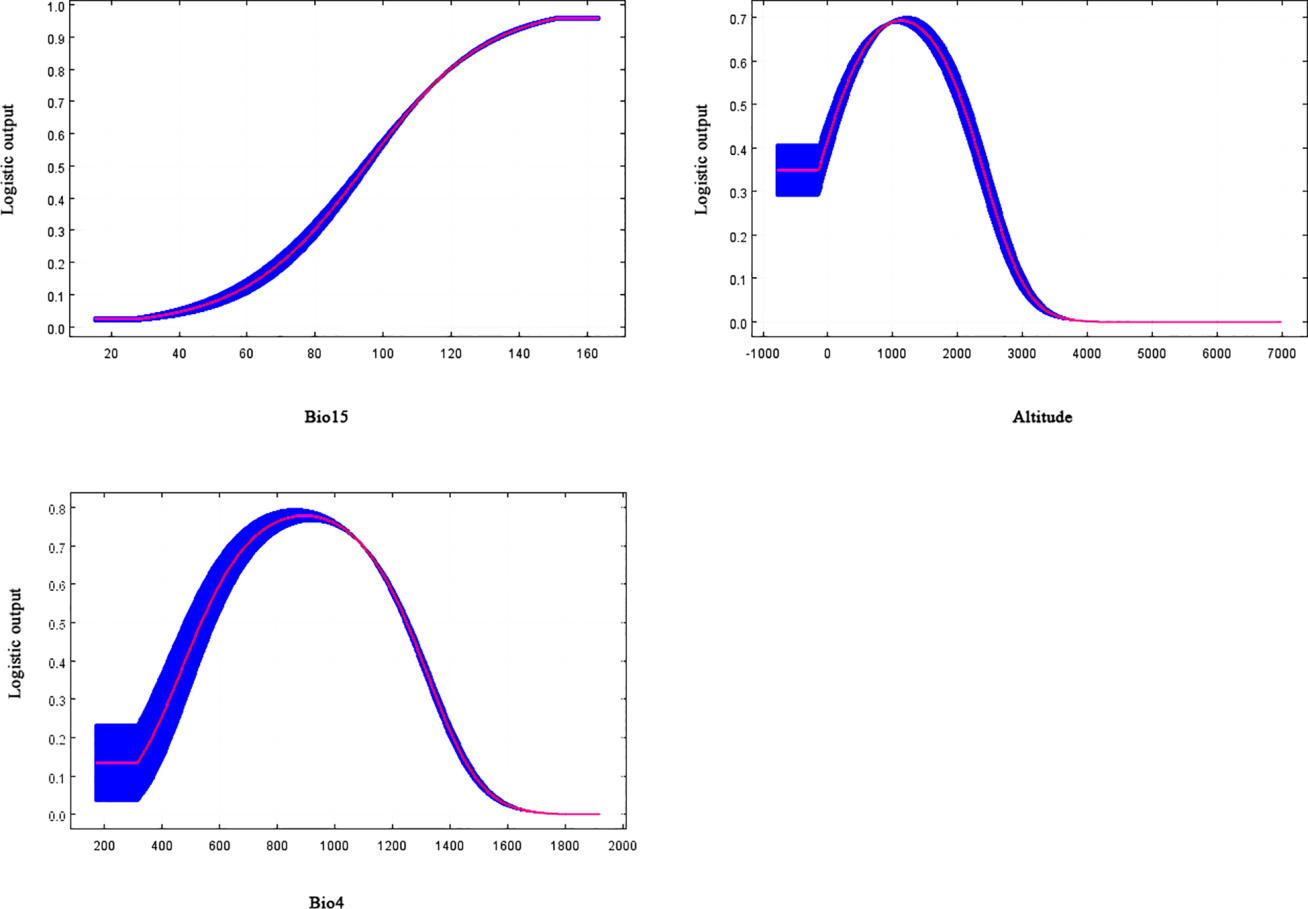
Response curve of naked oat existence probability to the main factors.

From the results of the response curve of naked oat existence probability to the main factors (**Figure 5**), for precipitation seasonality (coefficient of variation) (contribution rate 29.52%, replacement important value 16.09%), the survival probability of naked oat exceeded 0.5 from 96 and gradually increased, reaching a maximum value (0.96) at about 150, and then gradually stabilized. Therefore, the standard deviation suitable for the growth of naked oat was 95~150. With regard to altitude (contribution rate 25.76%, replacement important value 39.58%) from 200 m, the survival probability of naked oat was above 0.5 and began to increase, and when it reached about 1100 m, the survival probability reached the maximum value (0.57), and then began to decline rapidly. When the altitude was approximately 2100 m, the survival probability was reduced to 0.5; thus, the altitude range suitable for naked oat growth was 200 m–2100 m. When the temperature seasonality (contribution rate 11.95%, replacement important value 21.40%) was less than 540, the survival probability of naked oat was below 0.5. With an increase in the standard deviation, the survival probability of naked oat increased. At 1100, the survival probability of naked oat. The existence probability of oats was highest (0.77). With an increase in the standard deviation, the existence probability of naked oat began to decrease, and the survival probability began to reduce below 0.5 at approximately 1270. Therefore, the suitable standard deviation for the growth of naked oat was 1000–1230.

### Potential distribution areas of naked oat under modern climatic conditions

Among the 59 logical values of the presence probability of naked oat in the modern climate, the highest logical value was in Gu’an County, Langfang City, Hebei Province (0.95), the lowest logical value was in Inner Mongolia (0.05), and the average logical value was 0.55. The potential distribution areas of naked oat in the modern climate were mainly located in Shanxi, southwestern Hebei, north of Tianjin, and southern and southeastern Inner Mongolia.

**Figure 6:**
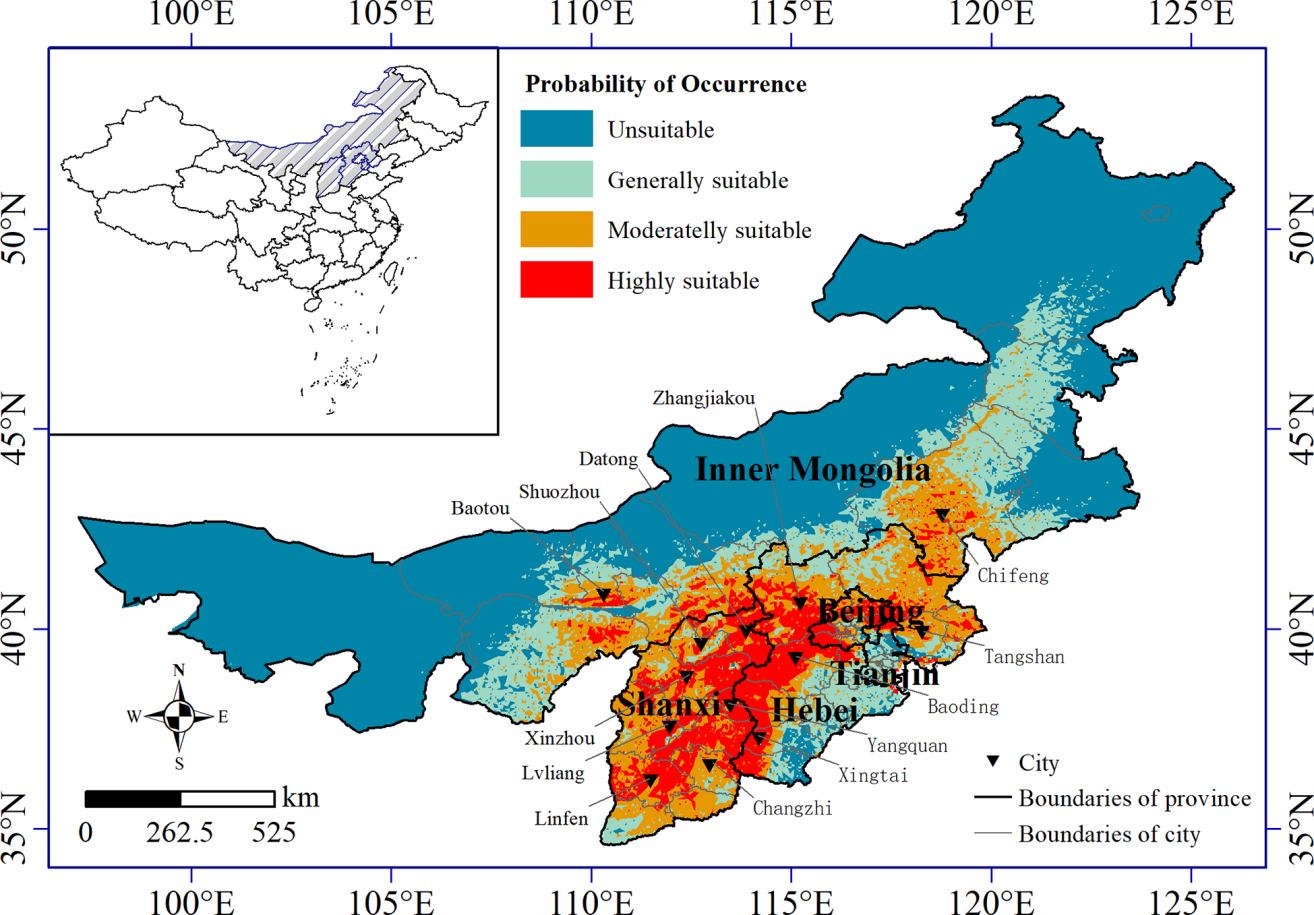
Suitable growing area for naked oat under modern climatic conditions.

The most suitable area for naked oat was 27.89×10^4^ km^2^, accounting for 17.91% of the total area in North China (**Table 3**). The most suitable distribution area for naked oat was mainly located in central and northeastern Shanxi, southwestern Hebei, and eastern Beijing. The highly suitable area for naked oat was 30.50×10^4^ km^2^, mainly located in southwestern and northwestern Shanxi, southwestern Inner Mongolia, and eastern and northern Hebei. The total suitable area for naked oat was 46.94×10 ^4^ km^2^, accounting for 30.15% of the total area in North China. From the Maxent model prediction results, the potential geographic distribution range of naked oat was much larger than the modern geographic distribution range of naked oat (**Figure 1**, **6**).

**Table 3:** The proportion of suitable areas for growing different grades of naked oat in North China.

Region	area(km^2^)(rate(%))			
	Unsuitable areas	Less suitable areas	Suitable areas	High suitable area
Beijing	0.00 (0.00%)	0.02 (0.01%)	0.03 (0.02%)	1.56 (1.00%)
Tianjin	0.42 (0.27%)	0.25 (0.16%)	0.20 (0.13%)	0.25 (0.16%)
Hebei	1.00 (0.64%)	2.99 (1.92%)	4.04 (2.60%)	10.26 (6.59%)
Shanxi	0.05 (0.03%)	0.81 (0.52%)	3.58 (2.30%)	10.46 (6.72%)
Inner Mongolia	48.29 (31.02%)	42.86 (27.53%)	22.65 (14.55%)	5.35 (3.44%)

### Projections of the impact of future climate change on the potential geographic distribution of naked oat

**Figure 7:**
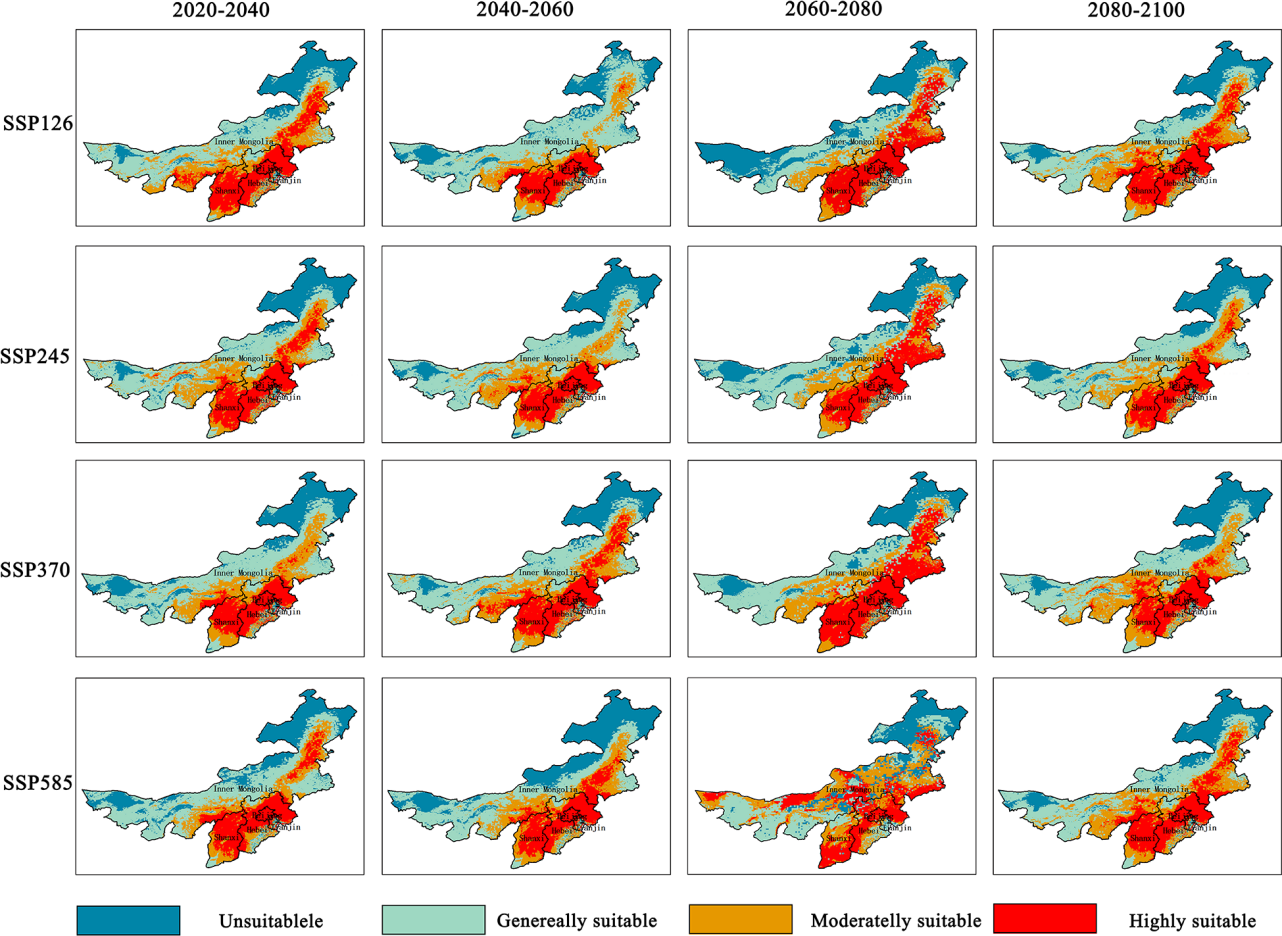
The potential distribution of naked oat under future scenarios.

We used the average of the prediction results of three GCM models to produce the climate suitable area distribution map, which was used to reduce the influence of choosing a single GCM model on the simulation results. **Figure 7** shows the change in the potential climate suitable area and range of naked oat in North China under different emission paths (SSP126, SSP245, SSP370, SSP585) in the future.

**Figure 8:**
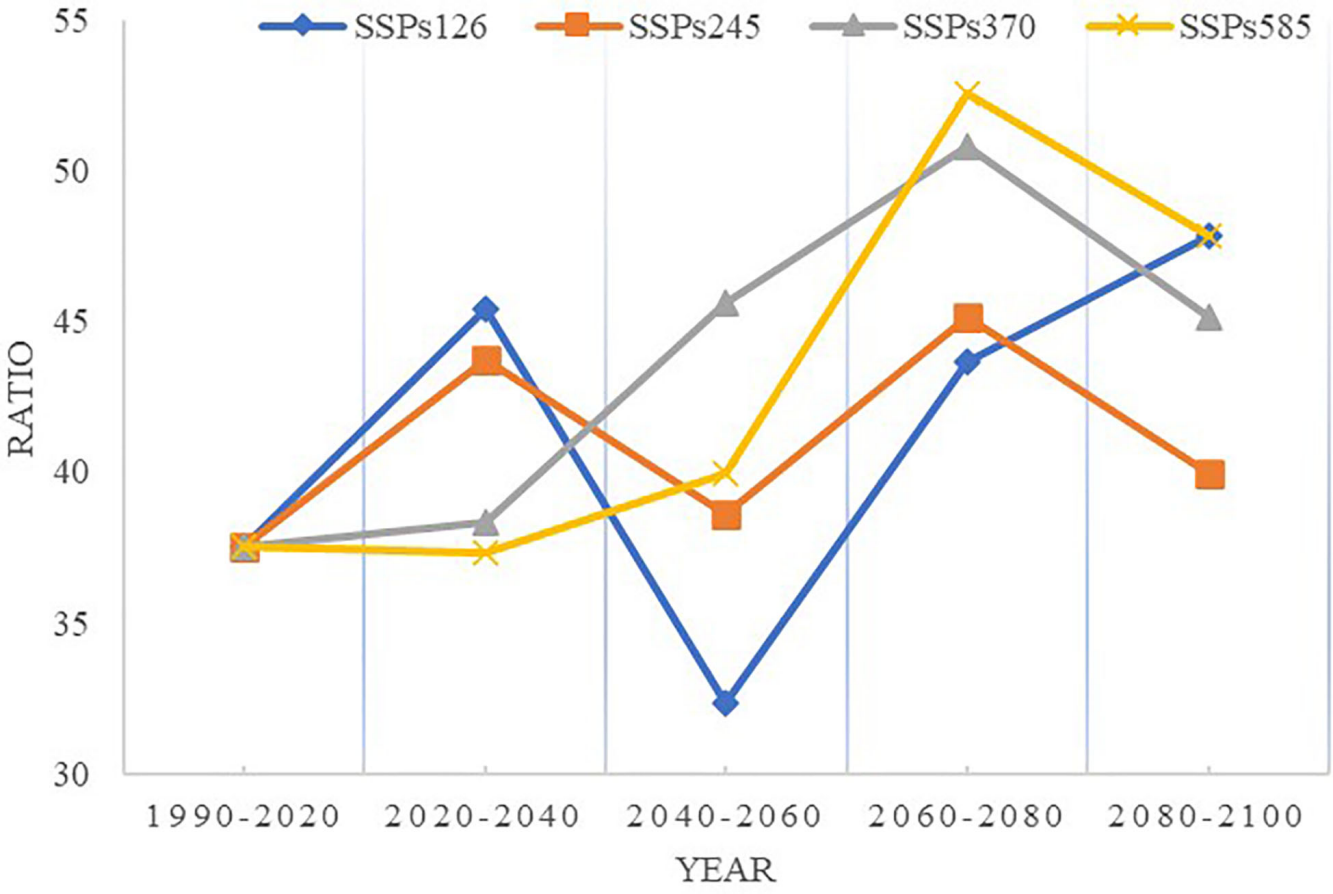
Changes in naked oat in the climate suitable area (habitat suitability>0.29) from 2020 to 2100 based on different shared socio-economic paths.

The suitable area for growing naked oat was designated as the suitable climatic zone of naked oat (highly suitable growing area and most suitable growing area). According to **Figure 8**, the area of climate suitable area of naked oat under the shared socio-economic path of SSP126 and SSP245 showed a decreasing trend from 2040 to 2060, especially under the shared socio-economic path of SSP126. Under the shared socio-economic path of SSP370 and SSP585, the climate suitable area of naked oat showed a steady expansion tendency from 2020 to 2080, and decreased after 2100.

## Discussion

By sharing of global species distribution data and the rapidly developing spatial analysis techniques, ecological niche models have been pioneered and applied in several fields of biodiversity conservation. Ecological niche models with correlation schemes could infer the ecological needs of species and predict the actual and potential distribution at different times and regional scale based on the distribution data of species and relevant environmental variables. The complex topography of North China has a marked influence on the study of the suitability distribution of naked oat. The Maxent model simulates the potential distribution of the species with high accuracy and confidence using the data of the geographical distribution points of the species (Phillips et al., 2006; Ma, 2013; Merow et al., 2013). Based on the geographical information and environmental variables of 59 valid points in North China, the Maxent model and spatial analysis technology were used to predict the suitability distribution profile of naked oat in North China.

In contrast to previous studies, this study goes beyond considering the relationship between CO_2_ concentration and climate, and explores the impact of scenario changes in greenhouse gas emissions on the geographic distribution of species under socio-economic changes and policy intervention. Shared socio-economic pathways (SSPs) can be used to predict greenhouse gas emission scenarios under different climate policies in 2100, which differ from representative concentration pathways that consider socio-economic, land-use effects on the development of regional climate change (Moss et al., 2010; O’Neill et al., 2016; Lu et al., 2020). Comparing these results with those of the naked oat suitable habitats in the current climate, this study provides a suitable stable habitat for the species under climate change.

### Constraints of climatic factors on the potential geographical distribution of naked oat

The prediction of the Maxent model showed that the important environmental factors limiting the potential geographic distribution of naked oat were temperature, precipitation factors and topographic factors. As North China is in an arid zone where precipitation stress tends to be more prominent than temperature stress, naked oat is mainly distributed in North China. The potential geographic distribution of naked oat is more dependent on precipitation-related environmental factors (Zhang et al., 2013). Temperature changes have an essential influence on the latitudinal migration of vegetation zones. Without considering the moisture limitation, the areas where plant growth is greatly affected by heat will have an effect on plant growth as global warming increases. This phenomenon indicates that although precipitation has a greater influence on the potential geographic distribution of naked oat than temperature, temperature also plays a non-negligible role in the factors governing the potential geographic distribution of naked oat (Xu and Xue, 2013).

Future data on other environmental factors such as vegetation cover, soil and land-use variables also have an impact on the potential geographic distribution changes of naked oat. Due to the difficulty in obtaining vegetation cover, soil and land-use data in future periods, the prediction of the potential geographic distribution of naked oat has not been included. Therefore, the prediction results cannot be precisely applied to plots, and must be combined with local soil and hydrogeological conditions in practical applications. The results of this study are the first step in macro planning and are an important foundation for the rational cultivation of naked oat in fine agriculture in the future.

Although the effectiveness of species distribution models has been strongly supported by the literatures, it should be emphasized that the models are only tools to simplify reality, and the prediction is subject to certain prerequisites. Therefore, the model only considers the climatic suitability of the species. However, the geographical distribution of the species is not determined by climate completely, the other influential factors are closely related to the geographical distribution. It is extremely complex and difficult to understand to establish a model that includes all the parameters which interact with the target species. So from a practical point of view, it is necessary to use the simplified and stable models (Chejara et al., 2010; Kong et al., 2019). As for maxent model, although it has many advantages. For example, maxent model can still have high prediction accuracy in the case of less sample data. However, maxent model still has many shortcomings. In this research, we only consider the impact of meteorological data, and do not consider the impact of human activities such as water conservancy and irrigation. Because the first thing to do is to make suggestions on the selection of farming areas which is suitable for growing for local farmers, it is proper to introduce more human activity factors on the basis of planting planning in the suitable areas.

### Changes in the potential geographic range of naked oat under future climate change scenarios

Under the four different social scenarios of SSP126, SSP245, SSP370 and SSP585, the potential distribution areas of oat increased or decreased in varying degrees. Under the SSP126 scenario, the area of the unsuitable zone continuously increased during 2060 to 2080. While the area of the low suitable area fluctuated greatly, the area continuously increased during 2040 to 2060 and decreased sharply during 2060 to 2080. Under the SSP245 scenario, the area of the high and the most suitable areas changed little. Under SSP370 scenario, the area of unsuitable zone and high suitable area was not obvious during 2080 to 2100. The area of low suitable area decreased gradually, and the area of optimal growth area increased gradually. Under the SSP585 scenario, the area of unsuitable zone, high adaptive area and optimal growth area changed little. And the area of low adaptive zone increased during 2080 to 2100, and decreased gradually in other periods. Comparing the area changes of future climate suitable areas under different social scenarios, the performance of SSP245 social scenarios is more stable than that of the other scenarios. Compared with previous studies using Maxent model to predict species suitable growth areas, this study not only considered the relationship between carbon dioxide concentration and climate, but also explored the impact of scenario changes of greenhouse gas emissions on the geographical distribution of species under socio-economic changes and policy intervention. The shared socio-economic path (SSP) can be used to predict greenhouse gas emissions under different climate policies during 2020 to 2100, which is different from the representative concentration path. This channel considers the impact of socio-economic and land use on regional climate change and development (Guo et al., 2018). Compared to the results of the suitable growth zone of naked oat under the current climate, the stable suitable growth zone of species under climate change can be obtained.

In this study, changes in the potential geographic distribution of naked oat in China under different future climate scenarios were analyzed based on the Maxent model. Compared with the current potential geographic distribution of naked oat, the potential geographic distribution of naked oat under four socio-economic scenarios in 2080 showed an increase in the area and it was concentrated in suitable areas. Under the 2080 SSPs126 socio-economic scenario, the most suitable area of naked oat was mainly located in Linfen, Yangquan, Zhangjiakou in Hebei and small parts of Dongsheng and Tangshan. The highly suitable areas were mainly located in Shuozhou, Chengde, Baoding, and Chifeng. The suitability of naked oat for survival in the basin of the Dahei River to the Shining River decreased, and the most suitable area in the western and northern parts of Inner Mongolia also decreased. Under the 2080 SSPs245 socio-economic pathway, the optimum habitat of naked oat was relatively concentrated, and was mainly located in the eastern part of Shanxi and western Hebei. The high fitness areas were mainly concentrated in Lishi, Xingtai and Jining. Under the 2080 SSPs245 scenario, the optimum zone in Inner Mongolia increased, and the suitability of naked oat in northern Hebei increased compared with modern climate conditions (Duan et al., 2020). The results showed that the potential habitat area of naked oat increased in both the RCP4.5 and RCP8.5 scenarios, but decreased in the optimum and high habitat areas in the high CO_2_ concentration (RCP8.5) scenario, which is different to the conclusion of this study.

Under the 2080 SSPs370 scenario, the growth suitability of naked oat in Baotou, Dongsheng and Jining in Inner Mongolia decreased. The growth suitability of naked oat in Xinzhou and Changzhi in Shanxi, Zhangjiakou in Hebei, and Chifeng in Inner Mongolia increased. In addition, the area of the potential geographic distribution of naked oat in the area of optimum growth was the largest under the four shared socio-economic paths in 2080. Under the 2080 SSP585 shared socio-economic paths, the potential geographic range of naked oat in the area from Lishi, Changzhi and Datong to Zhangjiakou increased. The increased precipitation in China under the RCP8.5 emission scenario was higher than that under the low-concentration emission scenario (Mccarty, 2001). These results indicate that the increased precipitation under the high-concentration emission scenario could reduce or resolve the limitation of species distribution by precipitation factors, while the increased precipitation under the low-concentration emission scenario could not reduce or resolve the limitation of species distribution by precipitation factors. The effective water available for species under the low-concentration emission scenario will decrease due to global warming. Therefore, the potential geographic distribution of species located at the desert edge may be partially lost under the low-concentration emission scenario, which may also account for the largest niche and increase in naked oat under the RCP8.5 emission scenario. This phenomenon is consistent with previous studies on climate change affecting crop distribution changes, which suggest that the innovative potential geographic range of species will shrink with a warming climate and have a tendency to move to higher elevations (O’Banion and Olsen, 2014; Lai et al., 2018).

## Conclusion

In this study, the Maxent model was used based on environmental, altitude and soil variables to predict the potential spatial distribution pattern of naked oat in North China. The changing trend and distribution range of major suitable climatic planting areas from 2020 to 2100 were predicted based on four different socio-economic scenarios of three GCM models. The results showed that the important environmental factors limiting the growth of naked oat were temperature (annual mean temperature, coldest month and lowest temperature) and precipitation (wettest monthly precipitation and driest seasonal precipitation), and that naked oat is suitable for growing in areas with the wettest monthly precipitation of 3–38 mm, annual mean temperature of 7.5–25.8°C and driest seasonal precipitation of 0–30 mm. This is compatible with the characteristics of cold and cool, and drought tolerance. Under future climate conditions, naked oat will further shift to higher altitudes and higher latitudes, and this change will be more drastic under the high radiative forcing scenario, which may reduce the consistency between the main naked oat production areas and suitable climate areas.

## Data availability statement

The original contributions presented in the study are included in the article/supplementary material. Further inquiries can be directed to the corresponding author.

## Author contributions

XG, CW, and WC analyzed the data. NJ and MF contributed to the materials, wrote and were responsible for the manuscript. All authors contributed to the article and approved the submitted version.

## Footnotes

1
https://www.cvh.ac.cn

2
http://www.nsii.org.cn

3
http://ppbc.Iplant.cn

4
http://www.worldclim.com

5
http://www.fao.org.

6
http://www.ngcc.cn/ngcc/html/1/index.html

7
https://biodiversityinformatics.amnh.org/

8
^8^ http://shiny.conabio.gob.mx:3838/nichetoolb2/

## References

[B1] Bellard Céline Bertelsmeier C. Leadley P. Thuiller W. Courchamp F. (2012). Impacts of climate change on the future of biodiversity. Ecol. Letters. 15 (4), 365–377. doi: 10.1111/j.1461-0248.2011.01736.x PMC3880584 22257223

[B2] Bonan G. Doney S. (2018). Climate, ecosystems, and planetary futures: The challenge to predict life in earth system models. Science 359 (6375), eaam8328. doi: 10.1126/science.aam8328 29420265

[B3] Bo X. Z. Shi X. Y. Zhao J. C. Lin Q. Shi M. X. Shang M. F.. (2021). Climatic suitability of naked oat (Avena nuda l.) planting in China based on MaxEnt model. J. China Agric. University. 26 (09), 1–10. doi: 10.11841/j.issn.1007-4333.2021.09.01

[B4] Cheng X. L. Ren L. L. Yangi X. L. Liu S. J. Tong R. Zhou M. (2016). CMIP5 multi-model prediction of spatial and temporal characteristics of temperature and precipitation in China and its sub-regions. Hydrology 36 (04), 37–43.

[B5] Chejara V. K. Kriticos D. J. Kristiansen P. Sindel B. M. Whalley R. D. B. Nadolny C. (2010). The current and future potential geographical distribution of hyparrhenia hirta. Weed Res. 50, 174–184.

[B6] Chen J. S. Liu J. L. Zhu R. F. Li J. K. Di G. L. Zhang Q.. (2019). Bioclimatic characteristics of sheepgrass distribution areas in China based on MaxEnt analysis. J. Grasslands. 27 (01), 35–42. doi: 10.11733/j.issn.1007-0435.2019.01.006

[B7] Constandinou S. Nikoloudakis N. Kyratzis A. C. Katsiotis A. (2018). Genetic diversity of avena ventricosa populations along an ecogeographical transect in cyprus is correlated to environmental variables. PloS One 13 (3). doi: 10.1371/journal.pone.0193885 PMC5846772 29529086

[B8] Duan Y. Z. Wang C. H. Wang H. T. Du Z. Y. He Y.M. Chai G. Q.. (2020). Prediction of potential geographic suitable habitats of salix spp. in China under different climates based on ecological niche models. J. Ecology. 40 (21), 7668–7680. doi: 10.5846/stxb201902210306

[B9] Elith J. Graham C. H. Anderson R. P. Dudík M. Ferrier S. Guisan A.. (2006). Novel methods improve prediction of species’ distributions from occurrence data. Ecography 29 (2), 129–151. doi: 10.1111/j.2006.0906-7590.04596.x

[B10] Elith J. Phillips S. J. Hastie T. Dudík M. Chee Y. E. Yates C. J.. (2011). A statistical explanation of MaxEnt for ecologists. Diversity distributions 17 (1), 43–57. doi: 10.1111/j.1472-4642.2010.00725.x

[B11] Fan S. Chen C. Zhao Q. Wei J. Zhang H.. (2020). Identifying potentially climatic suitability areas for arma custos (Hemiptera: Pentatomidae) in China under climate change. Insects 11 (10), 674. doi: 10.3390/insects11100674 33020387 PMC7600814

[B12] Giorgi F. (2019). Thirty years of regional climate modeling: Where are we and where are we going next? J. Geophysical Research: Atmospheres. 124 (11), 5696–5723. doi: 10.1029/2018JD030094

[B13] Guisan A. Graham C. H. Elith J. Huettmann F. Distri N. S. (2007). Sensitivity of predictive species distribution models to change in grain size. Divers. Distrib. 13, 332–340. doi: 10.1111/j.1472-4642.2007.00342.x

[B14] Guo Y. L. Li X. Zhao Z. F. and Wei H. Y. (2018). Modeling the distribution of populus euphratica in the heihe river basin, an inland river basin in an arid region of China. Sci. China (Earth Sciences) 61 (11), 1669–1684. doi: 10.1007/s11430-017-9241-2

[B15] Hu X. C. (2007). Study on the enzymatic activity of oats and its inhibition process in food processing (Yanglin: Northwest Agriculture and Forestry University of Science and Technology).

[B16] Huang D. Q. Zhu J. Zhang Y. C.. (2013). Uncertainties on the simulated summer precipitation over Eastern China from the CMIP5 models. J. Geophysical Research: Atmospheres. 118 (16), 9035–9047. doi: 10.1002/jgrd.50695

[B17] Huang R. Du H. Wen Y. Zhang C. Zhang M. Lu H.. (2022). Predicting the distribution of suitable habitat of the poisonous weed astragalus variabilis in China under current and future climate conditions. Front. Plant Sci. 13. doi: 10.3389/fpls.2022.921310 PMC9531759 36204071

[B18] Jiang R. Zou M. Qin Y. Tan G. Huang S. Quan H.. (2022). Modeling of the potential geographical distribution of three fritillaria species under climate change. Front. Plant Sci. 12. doi: 10.3389/fpls.2021.749838 PMC8784777 35082804

[B19] Jiang Z. H. (2013). Challenges and innovative development of land degradation control under global change. World Forestry Res. 26 (06), 1–4.

[B20] Kaufmann R. Zhou L. Myneni R. Tucker C. J. Slayback D. Shabanov N. V.. (2003). The effect of vegetation on surface temperature: A statistical analysis of NDVI and climate data. Geophysical Res. Lett. 30 (22), 2147. doi: 10.1029/2003GL018251

[B21] Kong W. Y. Li X. H. Zou H. F. (2019). Optimizing MaxEnt model in the prediction of species distribution. Chin. J. Appl. Ecology. 30 (06), 2116–2128. 10.13287/j.1001-9332.201906.029 31257787

[B22] Kumar S. Stohlgren T. J. (2009). Maxent modeling for predicting suitable habitat for threatened and endangered tree canacomyrica monticola in new Caledonia. J. Ecol. Natural Environ. 1 (4), 094–098.

[B23] Lai Y. Y. Feng J. M. Yuan Y. Y. (2018). Impact of climate change on the altitudinal distribution pattern of tropical plants in Nepal. J. Xinyang Normal Coll. (Natural Sci. Edition) 31 (2), 233–239. doi: 10.3969/j.issn.1003-0972.2018.02.012

[B24] Li X. Tian H. Wang Y. Li R. Song Z. Zhang F.. (2013). Vulnerability of 208 endemic or endangered species in China to the effects of climate change. Region. Environ. Change 13, 843–852. doi: 10.1007/s10113-012-0344-z

[B25] Li G. Q. Du S. Guo K. (2015). Evaluation of limiting climatic factors and simulation of climatically suitable habitat for Chinese sea buckthorn. PloS One 10, e0131659. doi: 10.1371/journal.pone.0131659 26177033 PMC4503660

[B26] Liu S. B. (2014). Study on water source of desert riparian forest poplar based on stable isotope technique (Urumqi: Xinjiang Agricultural University).

[B27] Lu Y. Zhao Q. Cheng L. Zhao L. Zhang H. Wei J.. (2020). The potential global distribution of the white peach scale pseudaulacaspis pentagona (Targioni tozzetti) under climate change. Forests 11 (2), 192. doi: 10.3390/f11020192

[B28] Ma Y. H. (2013). Predicting the geographical distribution range of mosses based on maximum entropy model (MaxEnt) and geographic information system (ArcGis) (ShangHai: Shanghai Normal University).

[B29] Ma B. Sun J. (2018). Predicting the distribution of stipa purpurea across the Tibetan plateau *via* the MaxEnt model. BMC Ecol. 18, 10. doi: 10.1186/s12898-018-0165-0 29466976 PMC5822641

[B30] Mccarty J. P. (2001). Ecological consequences of recent climate change. Conserv. Biol. 15 (2), 320–333. doi: 10.1046/j.1523-1739.2001.015002320.x

[B31] Merow C. Smith M. J. Silander J. A. (2013). A practical guide to MaxEnt for modeling species’ distributions: what it does, and why inputs and settings matter. Ecography 36 (10), 1058–1069. doi: 10.1111/j.1600-0587.2013.07872.x

[B32] Moss R. H. Edmonds J. A. Hibbard K. A. Manning M. R. Rose S. K. Vuuren D. P.. (2010). The next generation of scenarios for climate change research and assessment. Nature 463 (7282), 747–756. doi: 10.1038/nature08823 20148028

[B33] Nian L. L. Yang Y. B. Yi X. F. Qi L. Liu X. L. (2020). Analysis of the current research status of oats based on bibliometrics from 2010 to 2019. Pratacultural Sci. 37 (6), 1160–1173. doi: 10.11829/j.issn.1001-0629.2019-0511

[B34] Ning Y. Lei J. R. Song X. Q. Han S. M. Zhong Y. F.. (2018). Simulation of the distribution of potentially suitable habitats for the limestone endemic plant hainan anemone. J. Plant Ecology. 42 (09), 946–954. doi: 10.17521/cjpe.2018.0066

[B35] O’Banion M. S. Olsen M. J. (2014). Predictive seismically-induced landslide hazard mapping in oregon using a maximum entropy model (MaxEnt). NCEE 2014 - 10th U.S. National Conference on Earthquake Engineering: Frontiers of Earthquake Engineering. doi: 10.4231/D3C24QN8T

[B36] O’Neill B. C. Tebaldi C. Detlef P.. (2016). The scenario model intercomparison project (ScenarioMIP) for CMIP6. Geoscientific Model. Dev. 9, 3461–3482. doi: 10.5194/gmd-9-3461-2016

[B37] Parmesan C. (2006). Ecological and evolutionary responses to recent climate change. Annu. Rev. Ecol. Evol. Systematics 37, 637–669. doi: 10.1146/annurev.ecolsys.37.091305.110100

[B38] Perkins-Taylor I. E. Frey J. K. (2020). Predicting the distribution of a rare chipmunk (Neotamias quadrivittatus oscuraensis): comparing MaxEnt and occupancy models. J. Mammal. 101, 1035–1048. doi: 10.1093/jmammal/gyaa057 33033469 PMC7528646

[B39] Phillips S. J. Anderson R. P. Schapire R. E. (2006). Maximum entropy modeling of species geographic distributions. Ecol. Modelling.190 3), 231–259. doi: 10.1016/j.ecolmodel.2005.03.026

[B40] Phillips S. J. Miroslav Dudík. (2008). Modeling of species distributions with maxent: new extensions and a comprehensive evaluation. Ecography 31 (2), 161–175. doi: 10.1111/j.0906-7590.2008.5203.x

[B41] Qin Z. Zhang J. DiTommaso A. Wang R. L. Wu R. S. (2015). Predicting invasions of wedelia trilobata (L.) hitchc. with maxent and GARP models. J. Plant Res. 128 (5), 763–775. doi: 10.1007/s10265-015-0738-3 26045231

[B42] Shabani F. Kumar L. Ahmadi M. (2018). Assessing accuracy methods of species distribution models: AUC, specificity, sensitivity and the true skill statistic. Glob. J. Hum. Soc Sci. 18, 6–18.

[B43] Shao M. Fan J. Ma J and Wang L. (2022). Identifying the natural reserve area of cistanche salsa under the effects of multiple host plants and climate change conditions using a maximum entropy model in xinjiang, China. Front. Plant Sci. 13. doi: 10.3389/fpls.2022.934959 PMC9432852 36061800

[B44] Stocker T. F. (2013). Climate change. the closing door of climate targets. Science 18, 280–282. doi: 10.1126/science.1232468 23196911

[B45] Su C. F. Song. G. Xin Z. Y. Li X. N. (2007). The status and role of small grains in the regional economic development of ningxia. Rain Fed Crops. 27 (03), 246–247.

[B46] Wang W. Li Z. J. Zhang Y. L. Xu X. Q. (2021). Current situation, global potential distribution and evolution of six almond species in China. Front. Plant Sci. 12. doi: 10.3389/fpls.2021.619883 PMC8102835 33968095

[B47] Wei J. F. Peng L. He Z. Q. Lu Y. Y. Wang F.. (2020). Potential distribution of two invasive pineapple pests under climate change. Pest Manage. science. 76 (5), 1652–1663. doi: 10.1002/ps.5684 31724310

[B48] Wiens J. A. Stralberg D. Jong somjit D. Snyder M. A.. (2009). Niches, models, and climate change: Assessing the assumptions and uncertainties. Proc. Natl. Acad. Sci. 106 Suppl 2 (Suppl 2), 19729–19736. doi: 10.1073/pnas.0901639106 19822750 PMC2780938

[B49] Williams J. W. Jackson S. T. Kutzbach J. E. (2007). “Projected distributions of novel and disappearing climates by 2100 AD,” in Proceedings of the National Academy of Sciences of the United States of America, 104 (14), 5788–5742. doi: 10.1073/pnas.0606292104 PMC1851561 17389402

[B50] Wu Z. N. Hou X. Y. Ren W. B.. (2018). Prediction of potential suitable habitats of lentil in China in the context of climate change. J. Grasslands 26 (4), 898–906. doi: 10.11733/j.issn.1007-0435.2018.04.015

[B51] Xu M. H. Xue X. A. (2013). Analysis of the effects on climate warming on growth and phenology of alpine plants. In: Arid Zone Resour. Environ. 03), 139–143. doi: 10.13448/j.cnki.jalre.2013.03.026

[B52] Yang G. W. Yang Y. D. Zang H. D. Zhao B. P. HU Y. G.. (20222022). Effects of biofertilizer substituting synthetic nitrogen fertilizer on growth and yield of naked oat in semi-arid area. . J. Inner Mongolia Agric. University（Natural Sci. Edition） 43 (1), 5–10. doi: 10.16853/j.cnki.1009-3575.2022.01.002

[B53] Zhang L. (2015). Application of MAXENT maximum entropy model in predicting the potential distribution range of species. Biol. Bull. 50 (11), 9–12.

[B54] Zhang H. Zhao H. X. Wang H. (2020). Potential geographic distribution of poplar in China under future climate change scenarios based on maxent model. J. Ecology. 40 (18), 6552–6563. doi: 10.5846/stxb201906111232

[B55] Zhang N. Zhao B. P. Zhang Y. L. Ren P. Liu J. H. Wang Y.. (2013). Changes in antioxidant enzyme activities and other physiological characteristics of oat leaves under drought stress and comparison of drought resistance. Agric. Res. Arid Regions 31 (001), 166–171.

[B56] Zhao S. F. Tian C. Y. Wang Z.G., L,Y.X.. (2007). Current status of oat production and scientific research in China and future development direction. Rain Fed Crops 06), 428–431.

[B57] Zhou H. T. (2014). Effect of environment on the nutrient quality traits of naked oat cultivars. Chin. Acad. Agric. Sci. MA thesis.

[B58] Zhou H. T. Na X. D. Zang S. Y. Xie R. Fl.. (2018). Application of the maximum entropy (Maxent) model in species habitat studies. Environ. Sci. Manage. 41 (03), 149–151.

[B59] Zurell D. Zimmermann N. E. Gross H. Baltensweiler A. Sattler T. Wuest R. O. (2020). Testing species assemblage predictions from stacked and joint species distribution models. J. Bio. 47, 101–113. doi: 10.1111/jbi.13608

